# A study of the progression of damage in an axially loaded *Branta leucopsis* femur using X-ray computed tomography and digital image correlation

**DOI:** 10.7717/peerj.3416

**Published:** 2017-06-23

**Authors:** Zartasha Mustansar, Samuel A. McDonald, William Irvin Sellers, Phillip Lars Manning, Tristan Lowe, Philip J. Withers, Lee Margetts

**Affiliations:** 1 Research Centre for Modelling and Simulation, National University of Science and Technology, Islamabad, Pakistan; 2 School of Earth and Environmental Science, University of Manchester, Manchester, UK; 3 School of Materials, University of Manchester, Manchester, UK; 4 Department of Geology and Environmental Geosciences, College of Charleston, Charleston, SC, USA; 5 School of Mechanical, Aerospace and Civil Engineering, University of Manchester, Manchester, UK

**Keywords:** X-ray computed tomography, Digital image correlation, *Branta leucopsis*, Axial loading, Progressive damage, Stress–strain, Deformation mechanisms, Computer modeling, Constitutive, Modeling and simulation

## Abstract

This paper uses X-ray computed tomography to track the mechanical response of a vertebrate (Barnacle goose) long bone subjected to an axial compressive load, which is increased gradually until failure. A loading rig was mounted in an X-ray computed tomography system so that a time-lapse sequence of three-dimensional (3D) images of the bone’s internal (cancellous or trabecular) structure could be recorded during loading. Five distinct types of deformation mechanism were observed in the cancellous part of the bone. These were (i) cracking, (ii) thinning (iii) tearing of cell walls and struts, (iv) notch formation, (v) necking and (vi) buckling. The results highlight that bone experiences brittle (notch formation and cracking), ductile (thinning, tearing and necking) and elastic (buckling) modes of deformation. Progressive deformation, leading to cracking was studied in detail using digital image correlation. The resulting strain maps were consistent with mechanisms occurring at a finer-length scale. This paper is the first to capture time-lapse 3D images of a whole long bone subject to loading until failure. The results serve as a unique reference for researchers interested in how bone responds to loading. For those using computer modelling, the study not only provides qualitative information for verification and validation of their simulations but also highlights that constitutive models for bone need to take into account a number of different deformation mechanisms.

## Introduction

Bone has a complex three-dimensional (3D) geometry, both in terms of its external shape and its internal structure. Bone grows and remodels itself according to the mechanical environment it experiences (Wolff, 1892) and reflects a combination of influences. The functional requirements of a particular bone mimics the animal’s very own habitat (Wolff, 1892). Femora (thigh bone) support the transmission of load due to the weight of the owner’s body, which can be structurally thought of as a long beam-like cylinder. With this geometry, it will have a distinct head, neck and shaft adapted to maximise mechanical strength at minimum weight (Brassey et al., 2013a, 2013b). Bone is a hierarchical material comprising key building blocks at specific scales including nano-, micro- and macro-scales. This hierarchical arrangement of bone is responsible for controlling properties like deformation and toughness (Mellon & Tanner, 2012; Ural & Vashishth, 2014). Femoral bone consists of two distinct regions, cortical bone which constitutes the exterior denser part and cancellous or trabecular bone. This sponge-like part is made of struts as small as 100–200 μm in diameter (Ghanbari & Naghdabadi, 2009). Distribution of trabeculae in the femur is such that most of the bony trabeculae lie in the neck and tension–compression trabeculae lie in the medial cortex region. This forms an efficient system to withstand stresses/forces under locomotion or other load bearing scenarios (Currey, 2000; Nagarajaa, Couseb & Guldberg, 2005; Rudman, Aspden & Meakin, 2006; Tomáš, 2006). In a femur, the possible fracture prone part is called the *Trigomeum intenum femoris* or ‘Ward’s triangle,’ which is made of diminished density of trabeculae lying in the upper epiphyseal femoral neck (Nagarajaa, Couseb & Guldberg, 2005; Tomáš, 2006). ‘Interstitial lamella’ are also found within the trabeculae in the upper and the lower condyles respectively. During loading scenarios where load exceeds the ultimate strength of bone these interstitial lamella break apart through cement lines causing bone to fail (Nagarajaa, Couseb & Guldberg, 2005; Weiner & Wagner, 1998; Launey, Buehler & Ritchie, 2010). During this breakage, the cement lines in the haversian canals are the first ones to fail, which then spread across the lamellar region of osteons.

Vertebrate long bones are usually designed to be tough by nature. If we take a closer look into 3D structure of the bone, some of its functional toughness can be investigated using geometrical information. It is difficult to accurately observe the microstructure solely from 2D microscopy investigations (Quinta Da Fonseca, Mummery & Withers, 2005) for bone’s toughness and deformation mechanisms. Consequently, to fully observe and understand this pathway, including the deformation mechanisms and overall mechanical response during an axial loading test, the state-of-the-art imaging is required to study the evolution of structure in 3D. As a non-destructive imaging technique, X-ray computed micro-tomography enables the virtual reconstruction of a 3D ‘image.’ X-ray attenuation coefficient used in X-ray computed tomography can reveal detailed quantitative information about the evolution of deformed features in the internal structure of an object when subjected to mechanical loading (Maire & Withers, 2014).

However, if X-ray computed tomography can be studied along with the digital image correlation (DIC) (Bay, 2008) or digital volume correlation (DVC) (Bay, 2008; Bay et al., 1999), it can give a very clear insight into mapping the heterogeneous deformation within the bulk of microstructured materials (Brémand, 2008). This combination works by correlating successive images so as to infer the displacement vectors relating one to the other (Zhang & Wang, 2015; Wang et al., 2016). In order to measure and extract the displacements, speckle-like internal contrast to X-ray absorption is required. While for 2D, surface-mapping contrast can be introduced artificially by adding high attenuation contrast markers, which is not very easy in 3D. Fortunately, in many cases the material microstructure itself has sufficient inherent contrast, such as for the study of the uniaxial mechanical response of cellular polymeric foam structures (Roux et al., 2008; McDonald et al., 2009, 2011) and trabecular bone (Verhulp, Rietbergen & Huiskes, 2004).

There are many examples where X-ray computed micro-tomography and DIC have been used to examine 3D local trabecular strains for small cubes of trabecular bone in mammals (McDonald et al., 2011), rodents (Christena et al., 2012; Hardisty et al., 2010) and humans (Libertiaux, Pascon & Cescotto, 2011; Pan, Wu & Wang, 2012). Such cubic specimens have also been used to provide the geometry for accurate computer simulations of bone (Levrero-Florencio et al., 2016a, 2016b, 2017). This paper for the first time, presents a study of a whole *Branta leucopsis* (Barnacle goose) femur, subjected to incrementally applied loading to failure whilst being monitored through the entire loading regime by 4D imaging. The purpose of the study was to investigate the range of deformation mechanisms that occur under axial loading in a typical vertebrate long bone. This data can then be used as a reference data set for computer modelling in vertebrate long bones.

## Methods

This section describes the rationale behind the selection of the specimen. It then details the procedures followed in the preparation of the specimen and the scanning of the specimen. The tools and methodology used to interpret the results are also explained.

### Selection of vertebrate long bone

**Figure 1:**
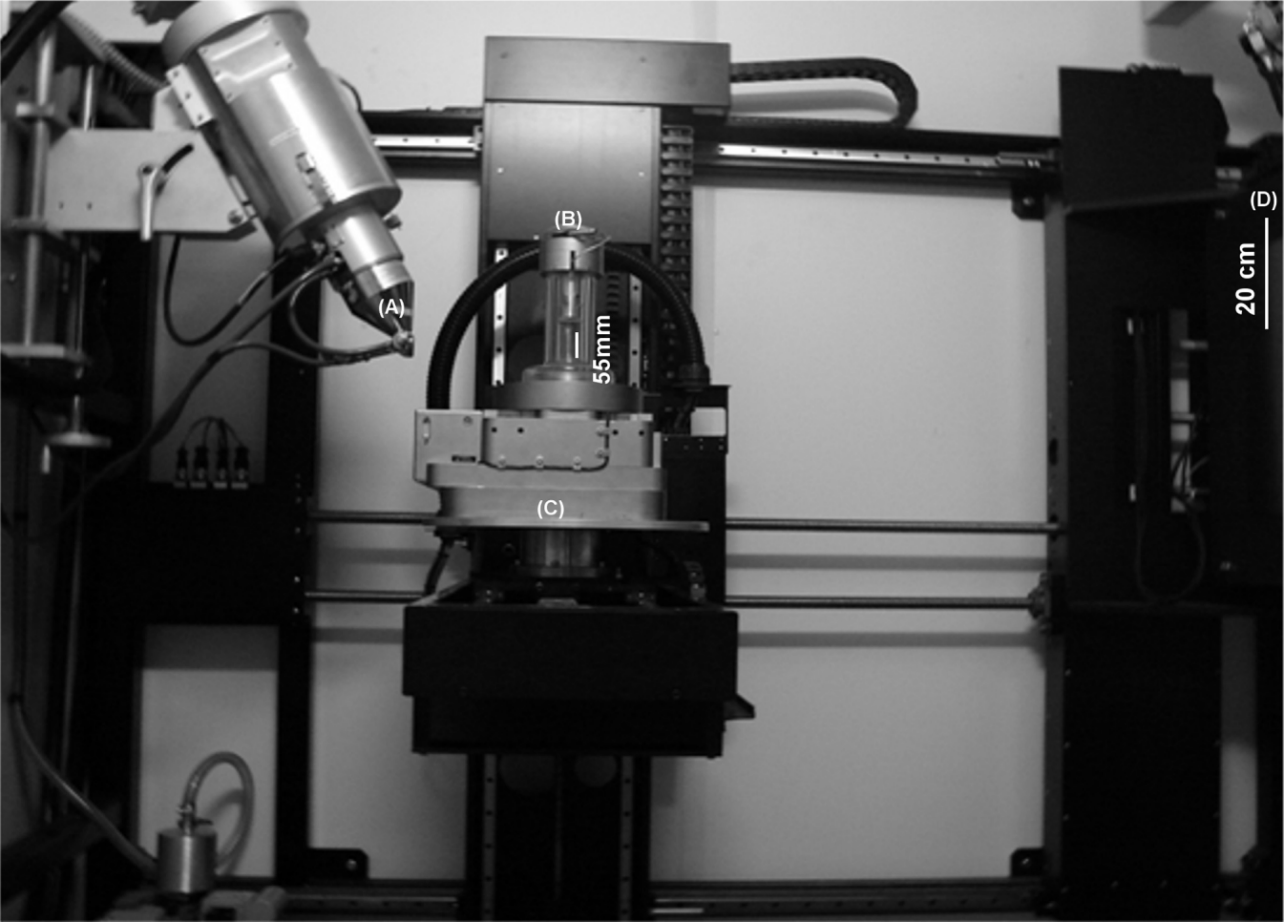
Nikon Custom Bay 320Kv X-ray micro-tomography system. The figure shows (A) the source, (B) the mechanical rig with the bone mounted for testing inside the perspex tube, (C) the load assembly and (D) the detector.

**Figure 2:**
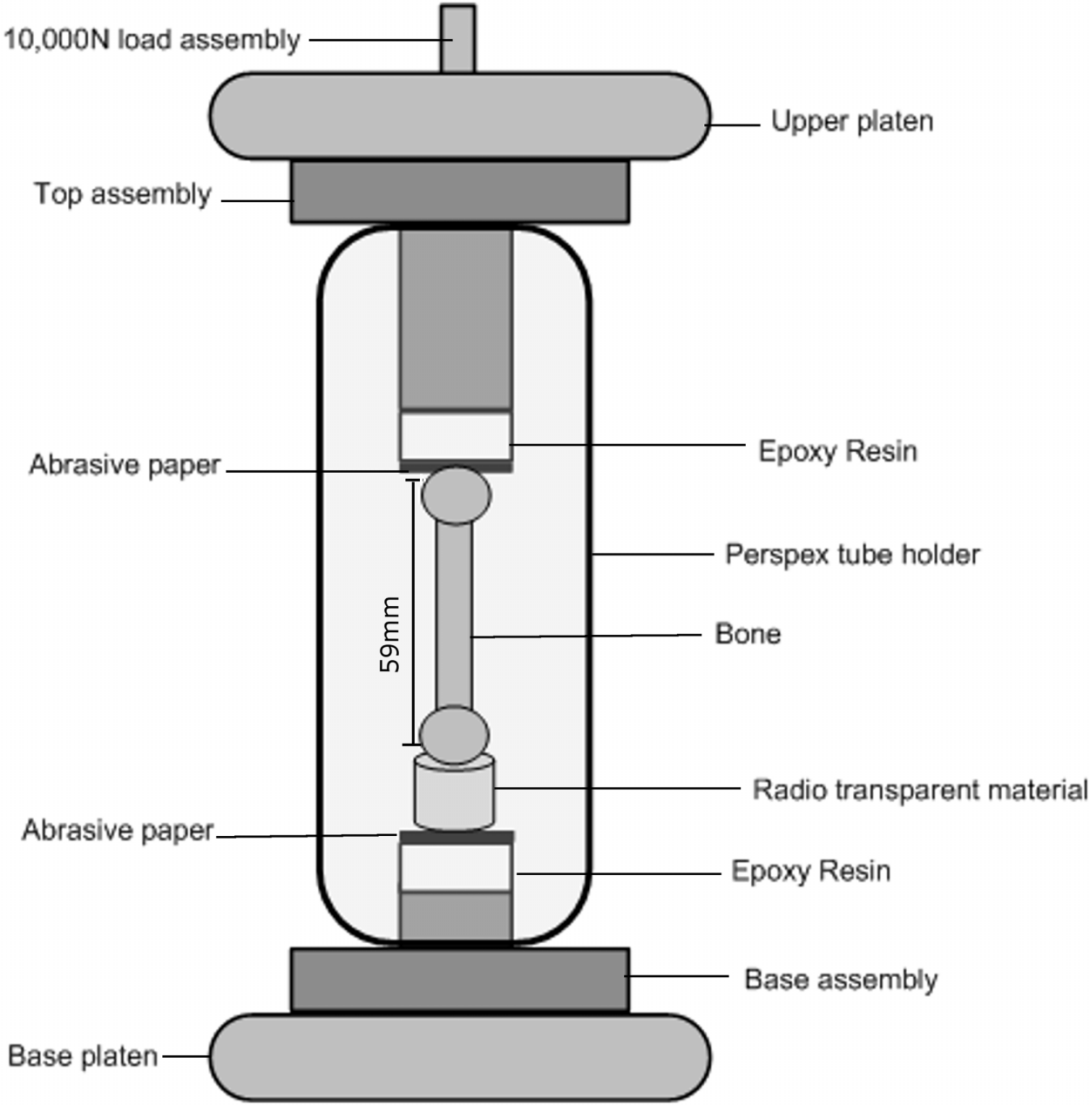
Loading cell. A schematic of the rig used for the mechanical compression test. The whole rig rotates on the X-ray instrument rotation table.

The characteristics of the test rig and scanning system placed some constraints on the size of bone and size of internal features under study. Simply stated, the bone cannot be longer than 70 mm so as to fit within the test rig (see Figs. 1 and 2) and only micro-structural features somewhat larger than one voxel can be captured. With respect to the latter, the high aspect ratio of long bones (long bones are significantly narrower than they are long) limits the resolution of scan that can be acquired.

Therefore, at the beginning of the study, a selection of bones from different species was identified as ‘test specimens’ on the basis of their lengths. The bones were individual femora from each of the following vertebrates: pigeon (*Columba livia*); partridge (*Perdix perdix*); Barnacle goose (*B. leucopsis*); Guinea fowl (*Numida meleagris*); pheasant (*Phasianus colchicum*) and rabbit (*Oryctolagus cuniculus*). These were sourced and prepared according to the procedure outlined in the next section.

A static X-ray tomography scan was carried out for each femur. Details of the scanning arrangements are given in the X-ray tomography section. The scans were reconstructed and the quality of each was assessed. In many of the specimens, micro-structural features in the cancellous bone were not captured as they were below the resolution of the scan. The goose bone produced a high-quality scan and the microstructure of the cancellous bone was very clearly defined. The goose was therefore selected for this study on the basis of its resolution and visibility of microstructure of the cancellous bone.

### Sample preparation

The right and left femora were obtained from an adult Barnacle goose (*B. leucopsis*) with accession number SF320. Both femora were 59 mm long. The intact adult weighed 2.03 kg. All soft tissues were carefully removed using standard laboratory procedures such that the distal and proximal epiphyses remained intact.

The bones were then cleaned following methodology of Cauble (2010) and Nawrocki (1997). This methodology entails taking care of the preparation of specimens such that the procedure does not weaken the bones before carrying out the mechanical test (Boyle, 2010; Grygon, 2010; Onwuama et al., 2012). All bones (including those in the previous section) were first treated with 75% saline solution to avoid use of organic/chemical. These bones were then subjected to ‘supervised boiling’ in tap water with a small amount of basic detergent for 6 h. During boiling the water was replaced after every hour. This released most of the soft tissue on the surface and the fats held within the marrow. Bones were then kept for air drying for three to four weeks. Drying was considered complete when the weight of the bone stayed constant over a few days.

The right femur was used as a ‘test specimen’ to devise, evaluate and improve the experimental protocols before carrying out the final destructive axial loading test on the left femur. Safety assessment procedures and protocols were followed in compliance with the BIOCOSSH procedures set out by the Medical School at the University of Manchester, UK (University of Manchester, 2016).

### X-ray tomography

A Nikon Custom Bay 320kV micro-CT scanner at the School of Materials, University of Manchester, UK (Fig. 1) was used for this study. The resolution acquired was 31.5 μm using 59 kV and 195 μA. The distance between the sample and the detector was 234 mm. In total, 2001 projections were collected over 360°. The acquisition time per scan was 50 min each. The number of pixels was 1,792 × 712 × 1,574. The pixel size was 0.0315 mm.

A specially designed mechanical loading rig was used with a scanner to carry out the axial loading. The rig has two adjustable steel platens, one on the top and the other on the base (see Fig. 1).

The left goose femur was mounted in the loading rig vertically along its axis. Epoxy resin disc spacers were specially manufactured and introduced between the bone and the steel platens at both the top and bottom of the loading rig. The purpose of the spacers was to avoid the steel platens casting a shadow on the tomography scans at the distal ends of the bone. Discs of abrasive paper of equal dimension to the epoxy resin were used between the bone and the resin in order to stop the bone moving laterally. Open cell phenolic foam (Smithers Oasis, Kent, OH, USA) was glued over the bottom epoxy resin disc, holding the bone upright. The foam is radio-transparent which avoids introducing any unwanted artefacts into the scan. The mounting protocols described were developed, tested and refined using other bone specimens. The loading rig and mounting of the specimen are shown in Fig. 2.

A 10,000 N load cell was used to load the bone through the upper platen. The lower platen was fixed. A compressive pre-load of 10 N was applied at the beginning of the experiment to take up any slack between the bone, the spacers and the rig. Once a stable force reading was reached (after ∼100–150 s), the load step was increased. In total 11 loading increments (using displacement control), numbered from 0 to 10 were applied at a rate of 0.5 mm/min. Loading was stopped when the bone had completely failed. The magnitudes of the displacements applied along with the corresponding loads are given in the results section. At the end of each displacement increment and when a stable force reading was acquired, a full 3D scan was carried out with the sample displacement fixed. The whole rig rotates on the X-ray instrument rotation table.

### Image processing

The images were reconstructed using the commercial CT-Metris Pro software (Nikon Metrology, Tring, UK). The centre of rotation was determined and the noise levels were reduced. Later on, the level of beam hardening was chosen. Data were exported in unsigned 16-bit DICOM format (VG Studio Max v. 2.0; Volume Graphics, Heidelberg, Germany). They were then processed using Avizo 7 (http://www.fei.com/software/avizo3d/). First a median filter, with a kernel size of 3×3×3 pixels, was applied in order to reduce the effects of noise.

The bone was segmented from the background using an interactive threshold tool in the Simpleware Ltd. software ScanIP (http://simplewareinc.com/wordpress/training-manuals/). The optimum threshold parameters were selected manually. Virtual isosurface renderings were then created for selected exemplars of the various deformation features (cracking, necking) found in the scans. Isosurfaces for each scan captured through the loading sequence were generated using the same thresholding parameters. This allowed the evolution of the features of interest to be followed from one scan to the next. The number of times each type of feature was present in each scan was counted manually to see whether there was a pattern of feature growth or feature emergence as the bone progressed to failure.

### Digital image correlation

Digital image correlation was used to evaluate the evolution of a small crack feature. This crack was chosen to be used for DVC, among the several deformation features reported in this study. Displacements were calculated in the 2D plane. Methods that have been developed include least-squares approaches (Roux et al., 2008) and Bayesian probability methods (Clocksin, Quinta Da Fonseca & Withers, 2002). In this study, correlation was performed using an algorithm developed by LaVision, Göttingen (originally conceived for particle image velocimetry (PIV)), which is part of the Strainmaster™ software package. It uses fast Fourier transform (FFT) cross-correlation to compare small sub-regions of the images (Quinta Da Fonseca, Mummery & Withers, 2005). The method is based on generalised texture mapping functions and on the fact that, under suitable conditions, groups of particles or regions of image texture will retain similar appearances under small translations and deformations. The particles or textured regions can then be tracked as a group using a correlation function to perform pattern matching as a function of the displacement. The algorithms thus use the characteristic local intensity variation to identify a region before and after deformation and thereby estimate the associated displacements. The actual displacement is determined from the location of the maximum value of the correlation function, and is always done to sub-pixel accuracy, with a local curve fit of the correlation data. If the correlations are performed correctly, the maximum value represents the most likely displacement of the image in each interrogation window. Multiple iterations are used during which the search sub-region is iteratively translated and deformed, using interpolation, until the highest correlation possible is achieved with accuracies of 0.01 pixels.

All the scans were imported into ImageJ (http://imagej.nih.gov/ij/). Scan 0, which is unloaded and therefore undeformed was selected as a reference image. The background was cropped for each image. Boundary pixels near the shaft in each image were aligned with reference to the first scan. All the images were then exported as a set of ‘raww’ files. These were imported into Avizo 7 and accurately aligned with one another using the affine registration module. DIC was then performed on the sequence of sub-images containing the crack feature. Correlation was performed on a sub-region of decreasing size, from 32×32 pixels to 16×16 pixels and with a sub-region overlap of 25%. This was found to provide a good compromise between spatial resolution and displacement accuracy, giving an uncertainty in strain resolution of around 0.05%. Strains were calculated by measuring the change in length/displacement between the original and the deformed images. The output was a sequence of strain maps corresponding to the associated loading increments.

## Results

The results are presented at three different levels of detail, defined here as macro-scale (the length scale of the whole bone), meso-scale (the length scale of individual trabeculae) and micro-scale (the length scale of crystalline lamellae that form the trabeculae). These correlate with the increasing fidelity provided by the different investigative tools employed. Firstly, the overall macro-scale load–displacement response is presented. Next, the authors present their study of the meso-scale deformation features captured by the X-ray tomography data. Finally, the results of the DIC are presented, giving insight into processes at the micro-scale.

### Macro-scale load–displacement response

**Figure 3:**
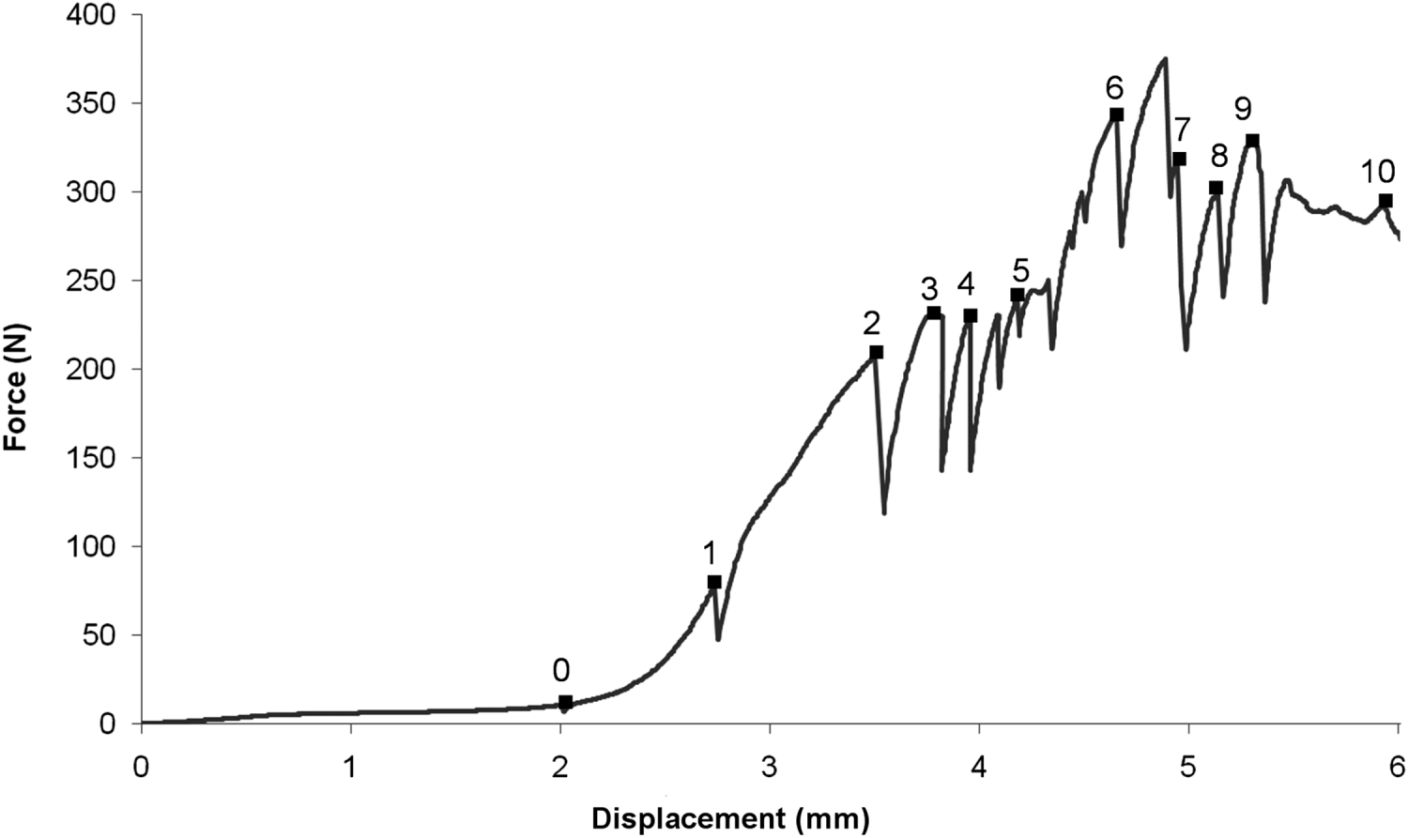
Load–displacement curve. Note that each scan label (0–10) plotted on the curve is located at the load–displacement reading taken just before carrying out the corresponding tomography scan (see Table 1).

**Figure 4:**
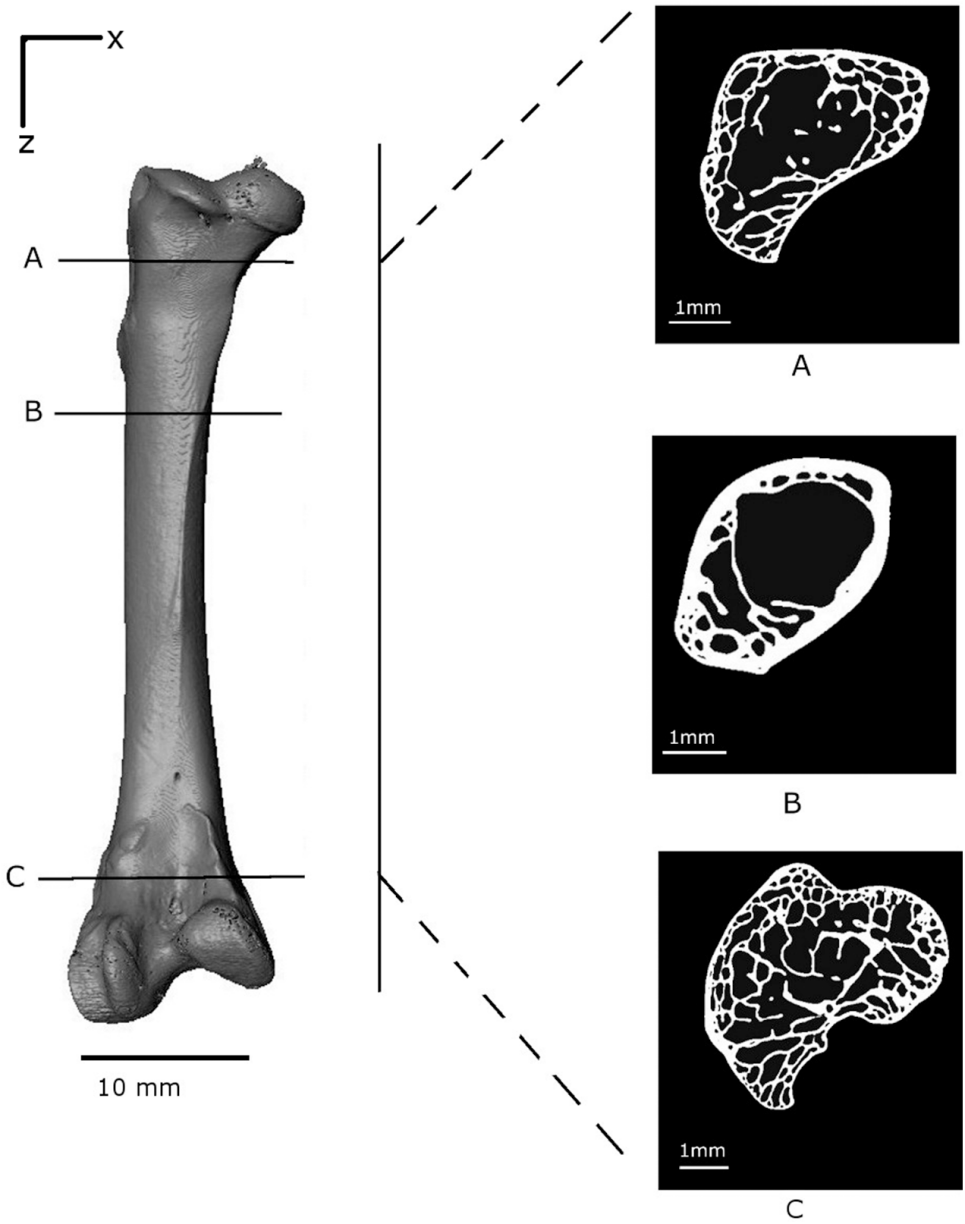
Volume reconstruction of the *Branta leucopsis* femur prior to loading (scan 0). The figure shows binarised cross-sections from the upper condyle (A), the shaft near the epiphyseal neck (B) and the lower condyle (C) to highlight the internal structure.

The load–displacement response is shown in (Fig. 3); the consecutive numbers ‘0’ to ‘10’ mark the points at which loading was interrupted to carry out the tomography scans. Whilst the scans were taken (∼50 min each), the loads decreased, probably due to creep in the bone material. The load–displacement data are also presented in Table 1. The first data point ‘0’ corresponds to the first tomography scan. A displacement of around 2 mm was recorded for an insignificant load reading. This represents the load required for all the slack to be taken up in the testing rig and for the bone to be held firmly in position. The reconstructed volume for the unloaded scan 0 is shown in Fig. 4.

**Table 1:** Force–displacement data for the *Branta leucopsis* femur.

Scan number	Force (Newton)	Displacement (mm)
0	10	2.04
1	77	2.73
2	208	3.50
3	230	3.75
4	229	3.96
5	250	4.24
6	343	4.65
7	247	5.02
8	296	5.11
9	310	5.25
10	273	6.02

Based on the load–displacement curve, there appear to be four stages in the macro- scopic response to loading. These are identified as follows: (i) The part of the curve from scan 0 to 2 appears to show an approximately linear load–displacement response; (ii) Scans 2–4 (displacement range 3.5–4.5 mm) corresponds to a plateaux (∼200–250 N) in the loading curve; (iii) Scans 5–6 show an increase in load (to ∼343 N) for a small increase in displacement (∼0.5 mm).

This is matched by the macro-scopic observation of a visible crack in the greater trochanter region of the head of the femur. (iv) Finally, scans 7–10 show a reduction in load bearing capacity of the bone which corresponds with shear failure in the epiphyseal region of the femur.

**Figure 5:**
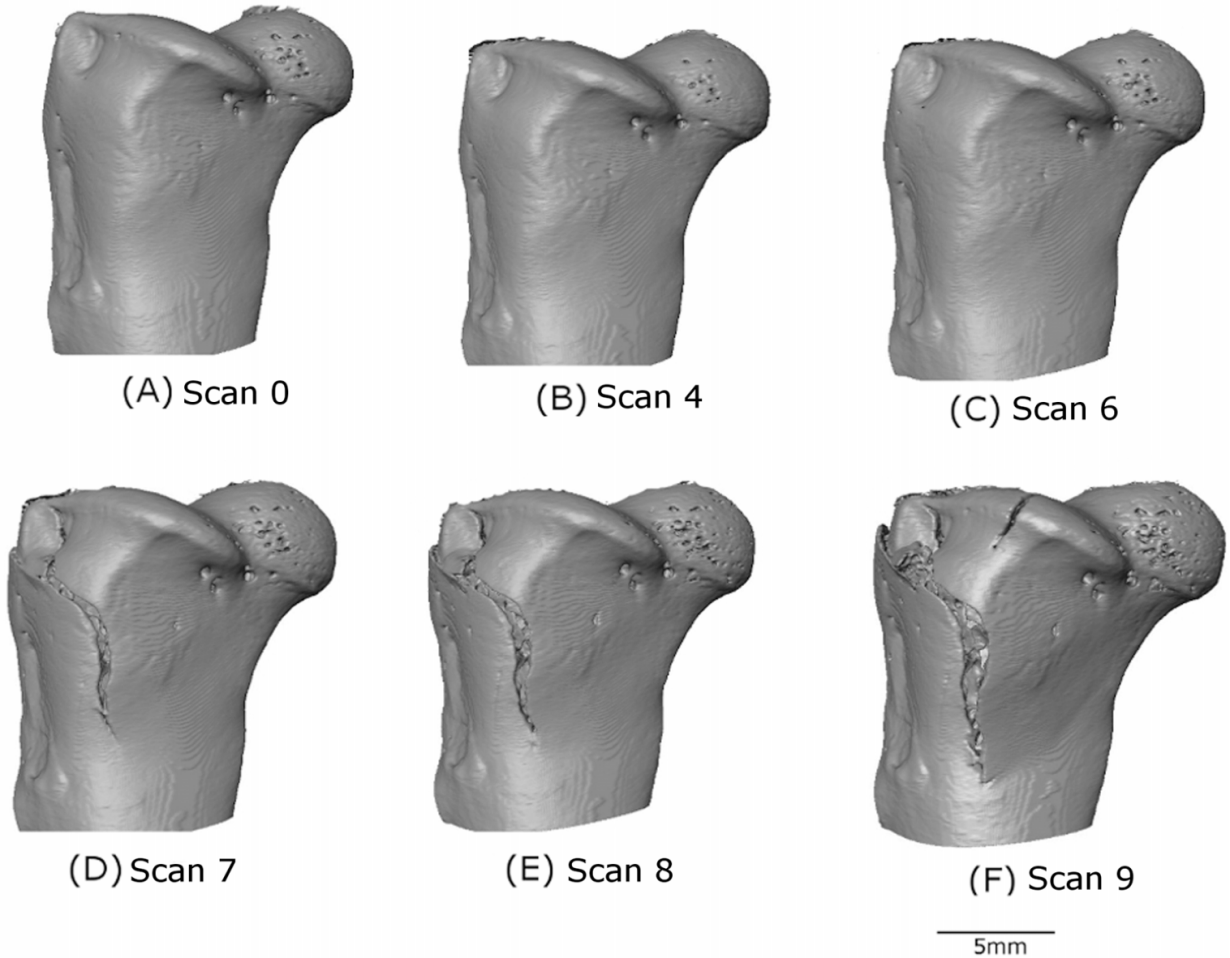
3D volume reconstruction of the macro-scale cracking. The evolution of the macro-scale crack on the upper part of the femur is illustrated for scans 0 (A), 4 (B), 6 (C), 7 (D), 8 (E) and 9 (F).

**Figure 6:**
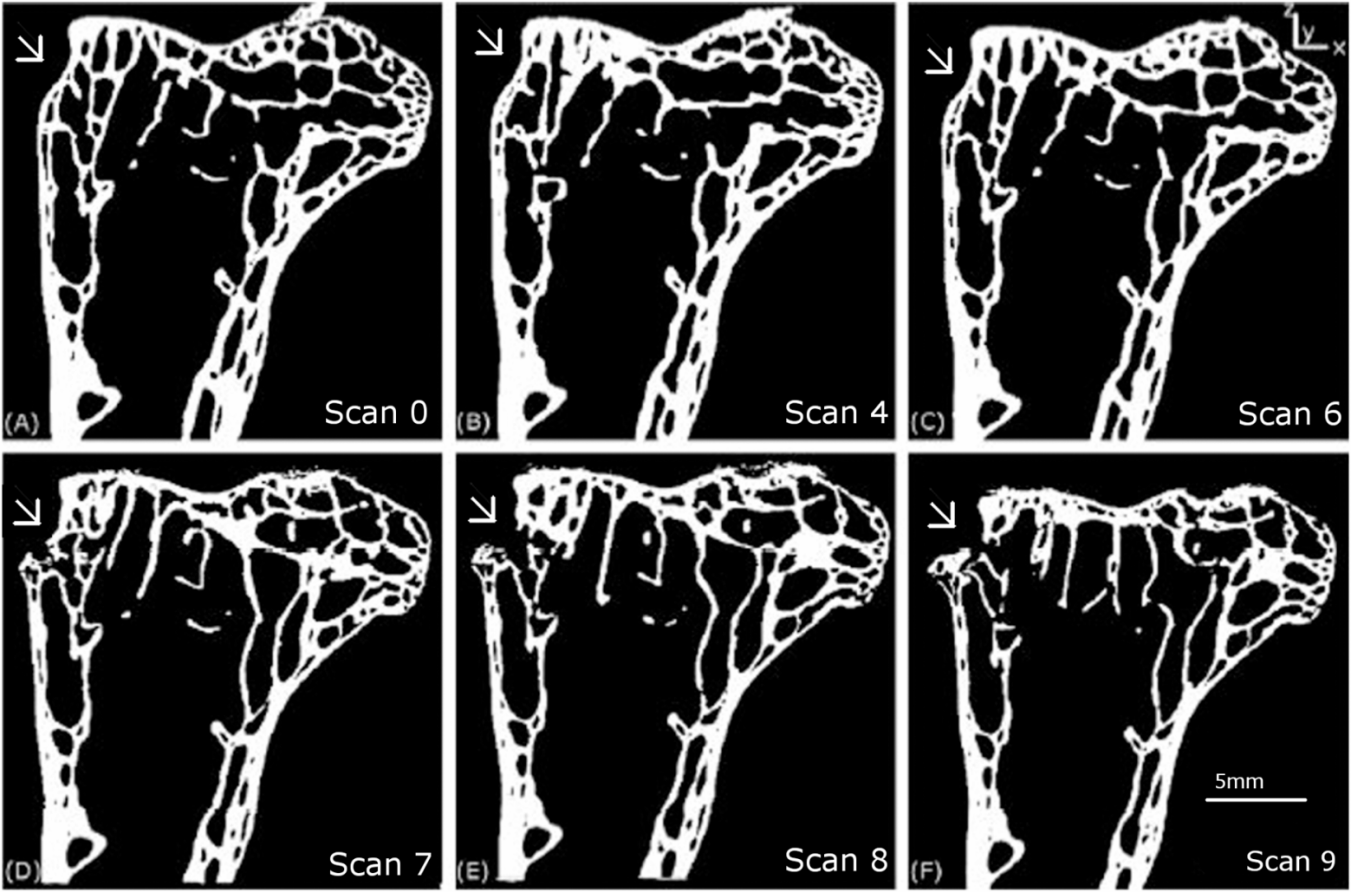
Longitudinal 2D tomography slices displaying the macroscopic crack. The evolution of the macroscopic crack on the upper part of the femur is shown for scans 0 (A), 4 (B), 6 (C), 7 (D), 8 (E) and 9 (F).

Figure 5 shows the external surface view (the macro level) of the progression of the crack and Fig. 6 shows 2D slices of the tomography scan for the same set of features from scan 0 to 9. These results are similar to those described in Ritchie (1988).

### Meso-scale deformation features

**Figure 7:**
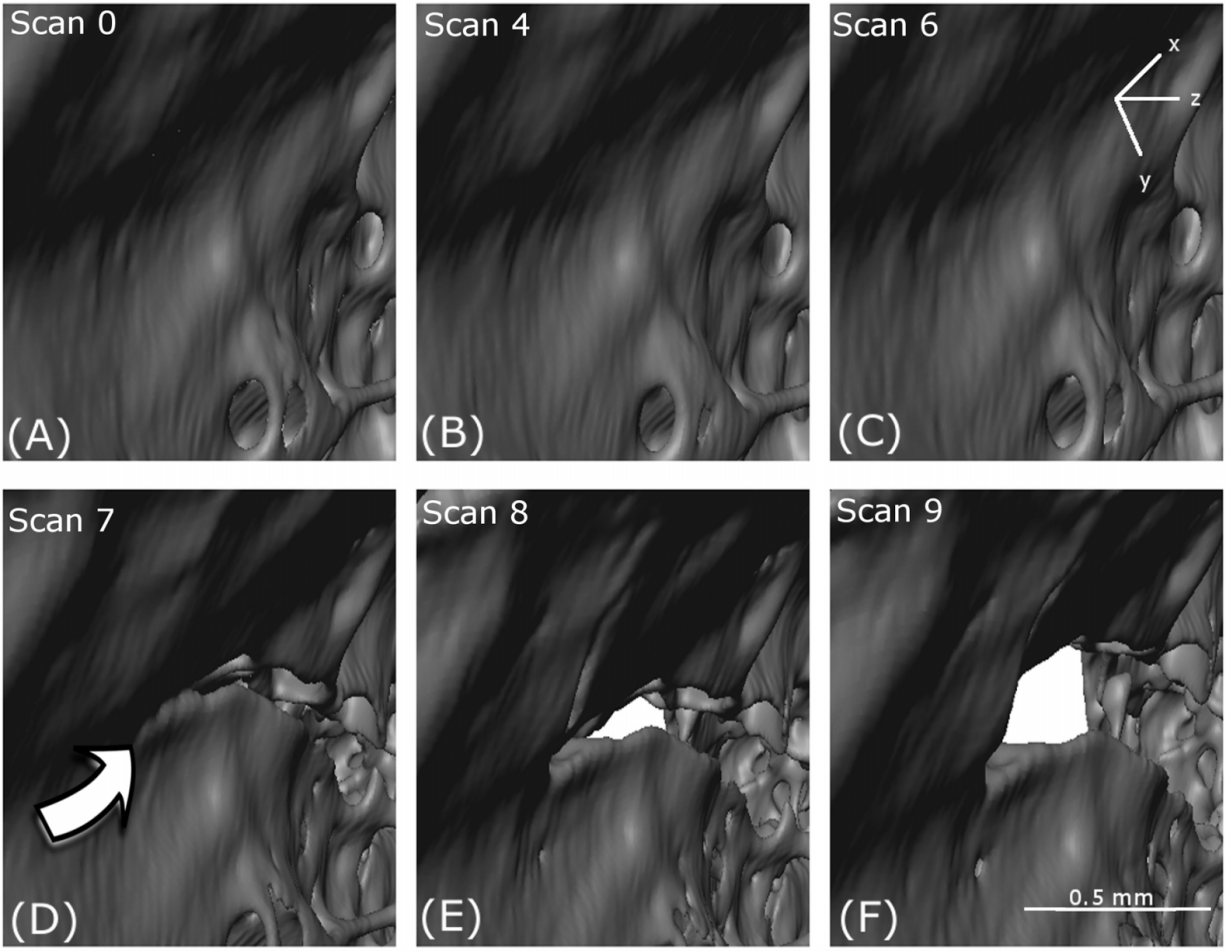
Localised crack opening and crack extension. (A–F) show reconstructed volumes from scan 0, scan 4, scan 6, scan 7, scan 8 and scan 9, respectively. The axis of compression is the *z*-direction as indicated.

**Figure 8:**
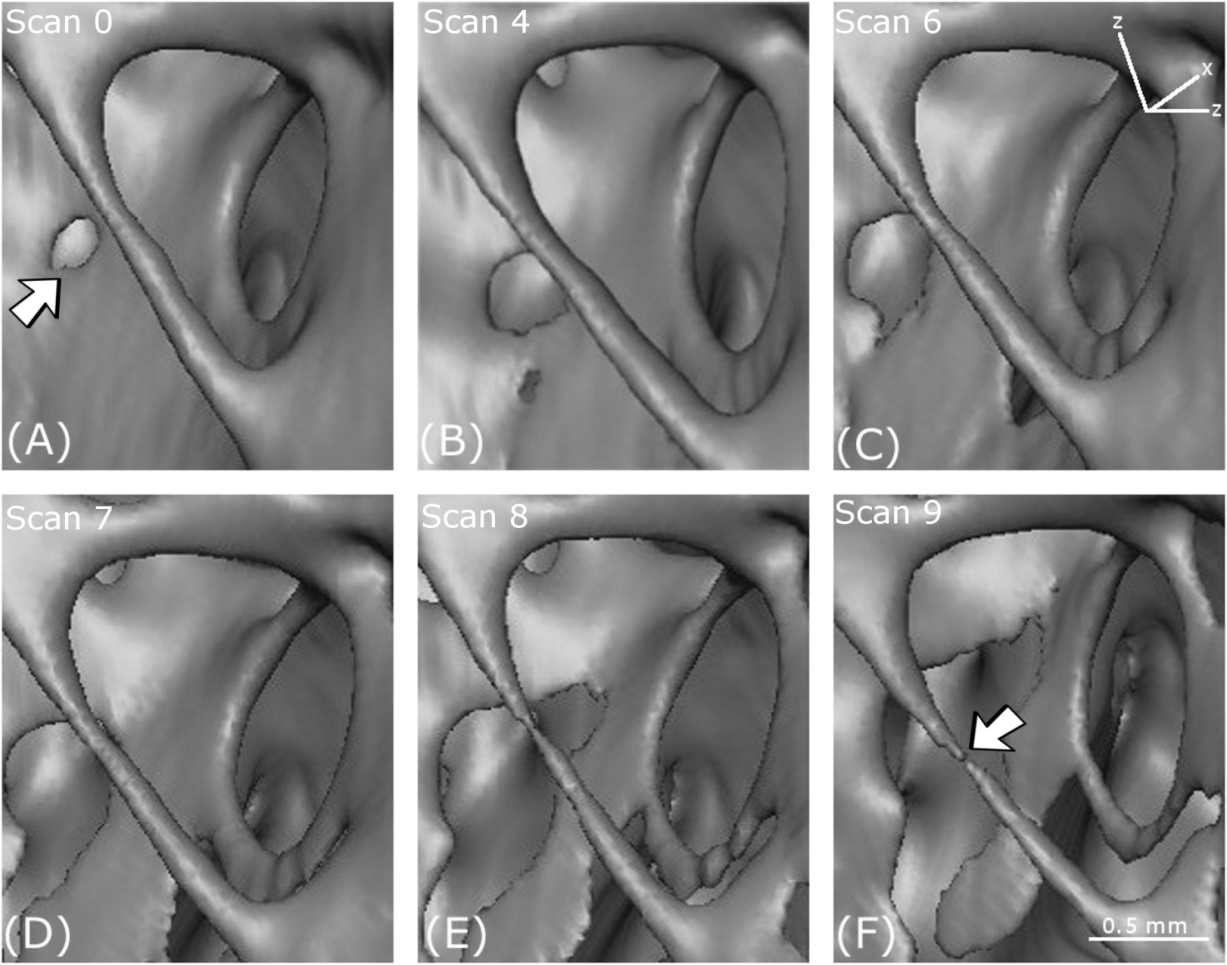
Thinning and tearing. Thinning and tearing of a cell wall and necking in a strut. (A–F) show reconstructed volumes from scan 0, scan 4, scan 6, scan 7, scan 8 and scan 9, respectively.

**Figure 9:**
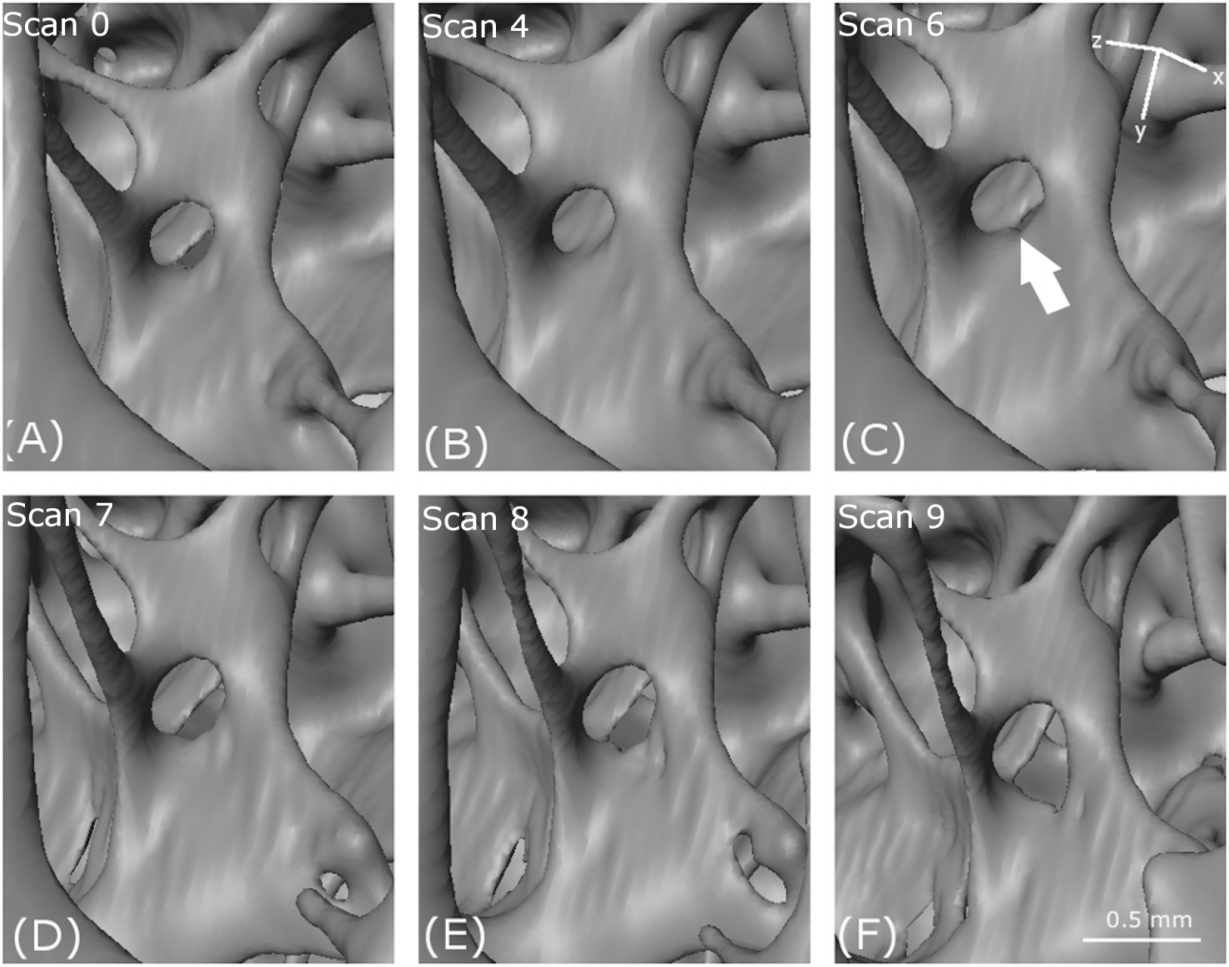
Notch formation. (A–F) show reconstructed volumes from scan 0, scan 4, scan 6, scan 7, scan 8 and scan 9, respectively. The location of the notch in the perimeter of the hole is indicated by the arrow in (C).

**Figure 10:**
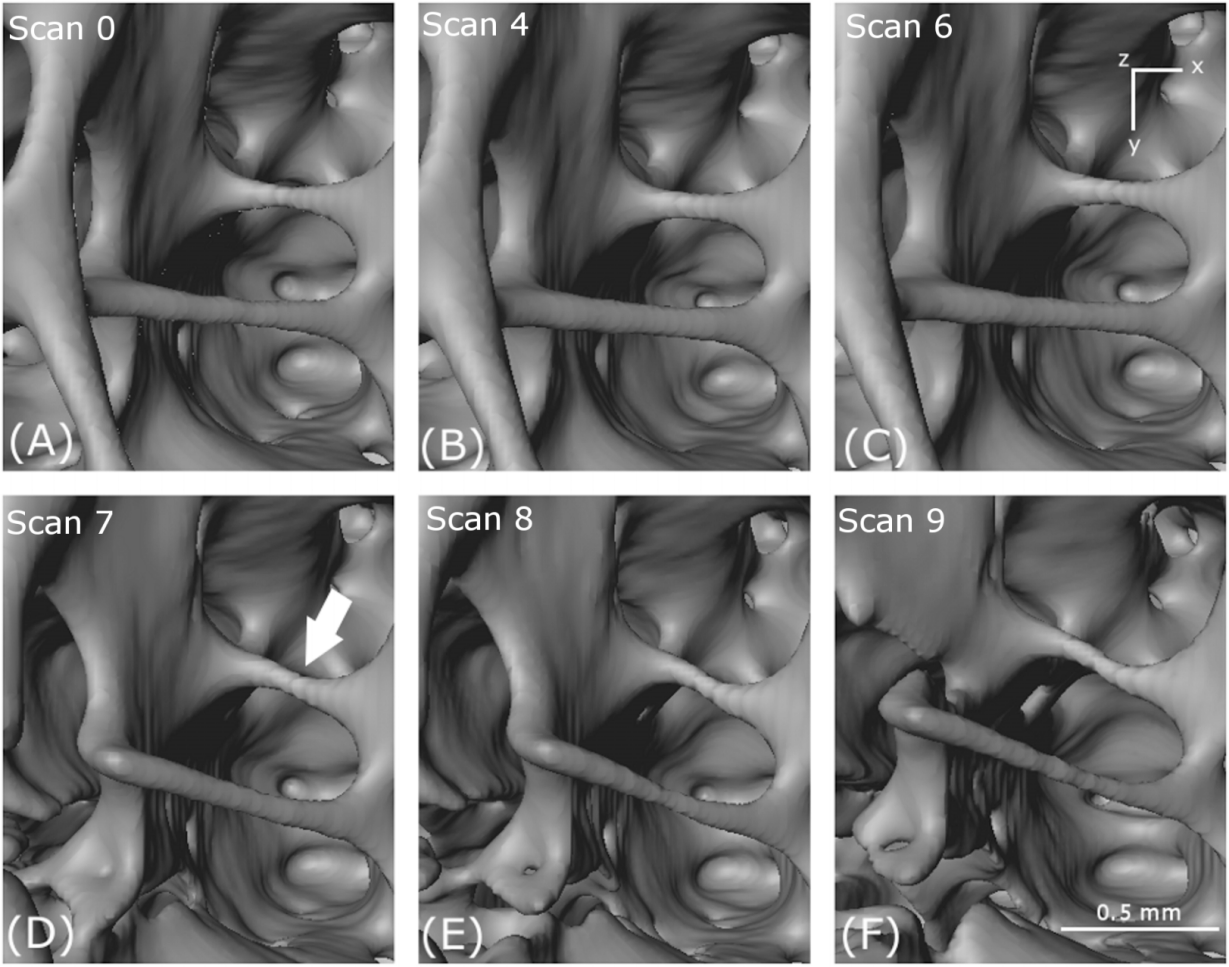
Buckling of struts. (A–F) show reconstructed volumes from scan 0, scan 4, scan 6, scan 7, scan 8 and scan 10, respectively. The strut undergoing buckling is indicated by the arrow in (D).

Five distinct types of deformation mechanism in the cancellous bone were identified from the reconstructed tomography data. Each of these appeared at specific points in the loading path. The evolution of these features could be followed to a certain degree at subsequent loading intervals. Both brittle and ductile mechanisms were observed, reflecting the varied influence of bone’s complex architecture. The mechanisms observed included (i) cracking (Fig. 7); (ii) thinning and (iii) tearing of cell walls and struts (Fig. 8); (iv) notch formation (Fig. 9) and (v) buckling of struts (Fig. 10). All these deformation features (Figs. 7–10) were located in the upper condoyle near the greater trochanter of the femoral head.

#### Cracking

A crack appears at scan 7 (D) in Fig. 7 which opens through scan 8 (E) and scan 9 (F). However, no obvious crack initiation mechanism is visible in the preceding scans. Cracking is examined more closely using DVC later in the paper.

#### Thinning and tearing

In Fig. 8, an essentially circular hole in a cell wall in the first scan (arrowed) increases in size with increasing loading. The increase in size is accompanied by cell wall thinning, leading to the creation of a new hole (scan 4) and tearing as the holes coalesce through (scan 6) and (scan 7). In the foreground, a strut thins (scan 7), then necks (scan 8) and eventually breaks (scan 9).

#### Notch formation

A notch appears in the perimeter of an approximately circular hole in a cell wall in Fig. 9. The notch is a stress concentrator that could lead to cracking. The formation of the notch is probably governed by mechanisms occurring in the crystalline microstructure that exists at a lower-length scale.

#### Buckling

An example of buckling is shown in Fig. 10 and is a classic mode of elastic deformation.

**Figure 11:**
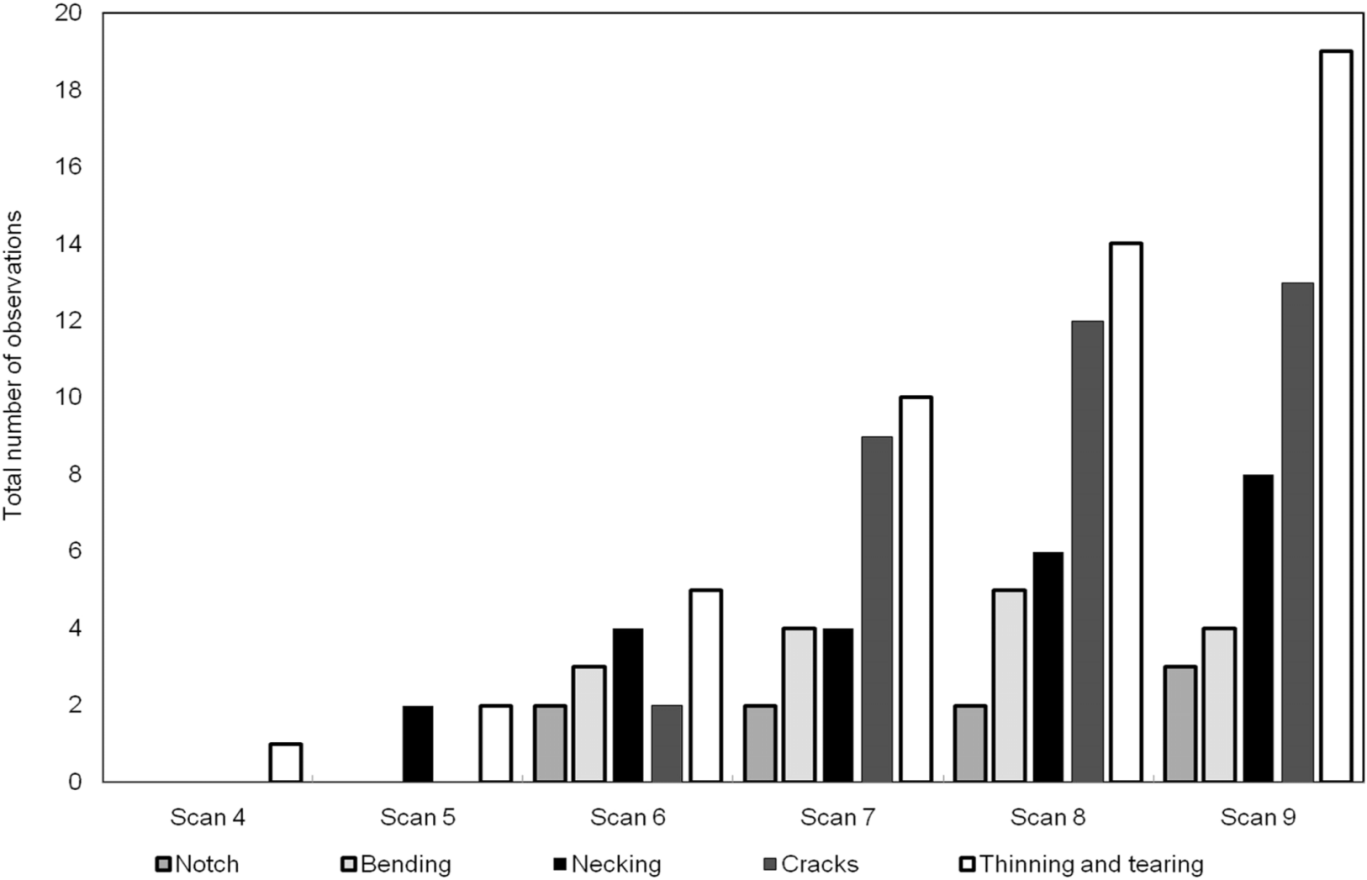
Histogram showing the number of each of the deformation features through the scans.

The number of occurrences of each of the meso-scale deformation features is plotted in Fig. 11 for the series of scans. The features rank from most to least dominant as follows: (1) thinning and tearing, (2) cracking, (3) necking, (4) bending and (5) notch formation.

### Micro-scale deformation

**Figure 12:**
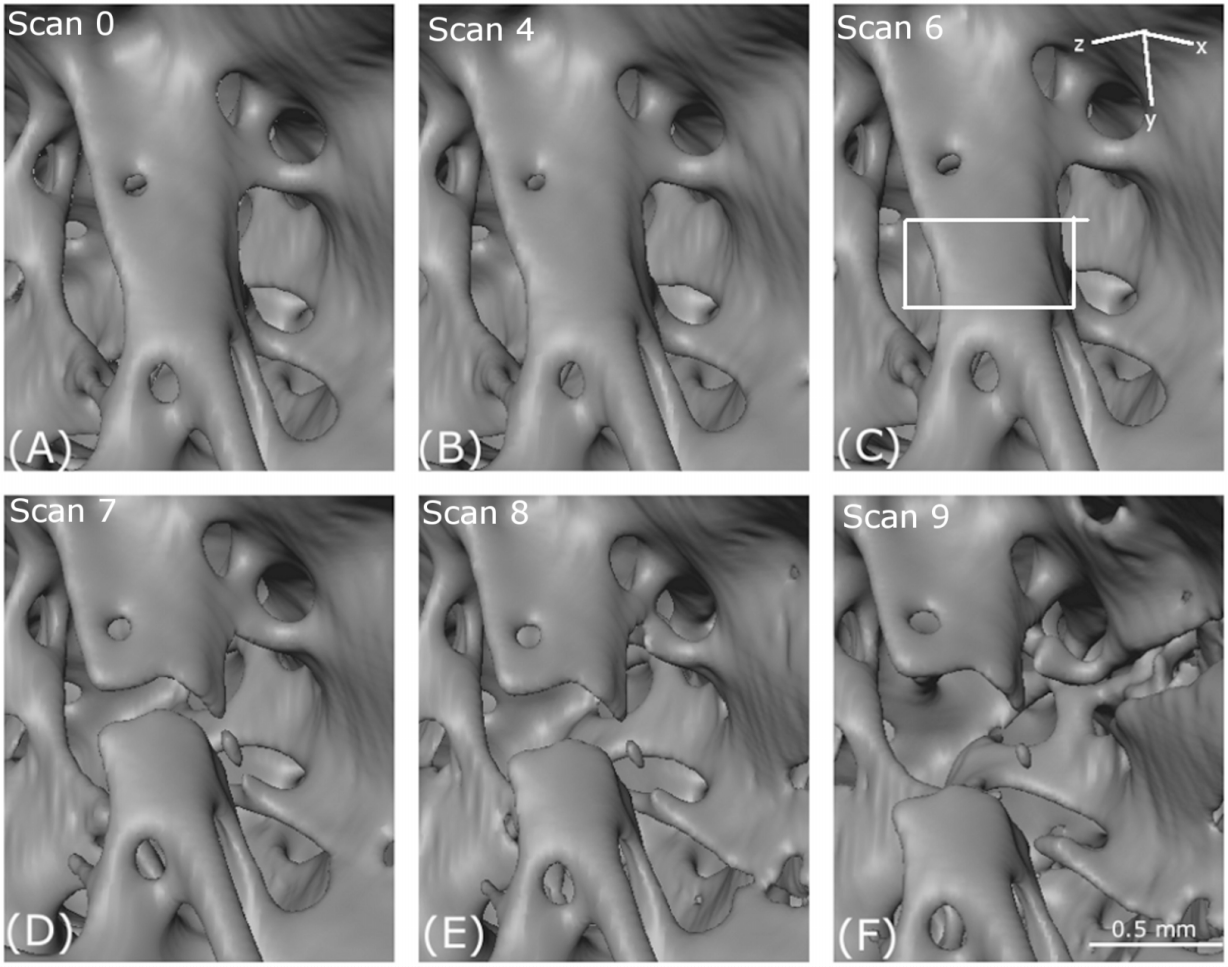
Crack propagation. Evolution of the failure of a trabecular strut. (A–F) shows incremental load steps from scan 0, scan 4, scan 6, scan 7, scan 8 and scan 9 (detailed data from Table 1). This crack was visible by eye from the external morphology of bone. The loading axis is in the *z*-direction. The white rectangular box indicates the region chosen for digital image correlation.

**Figure 13:**
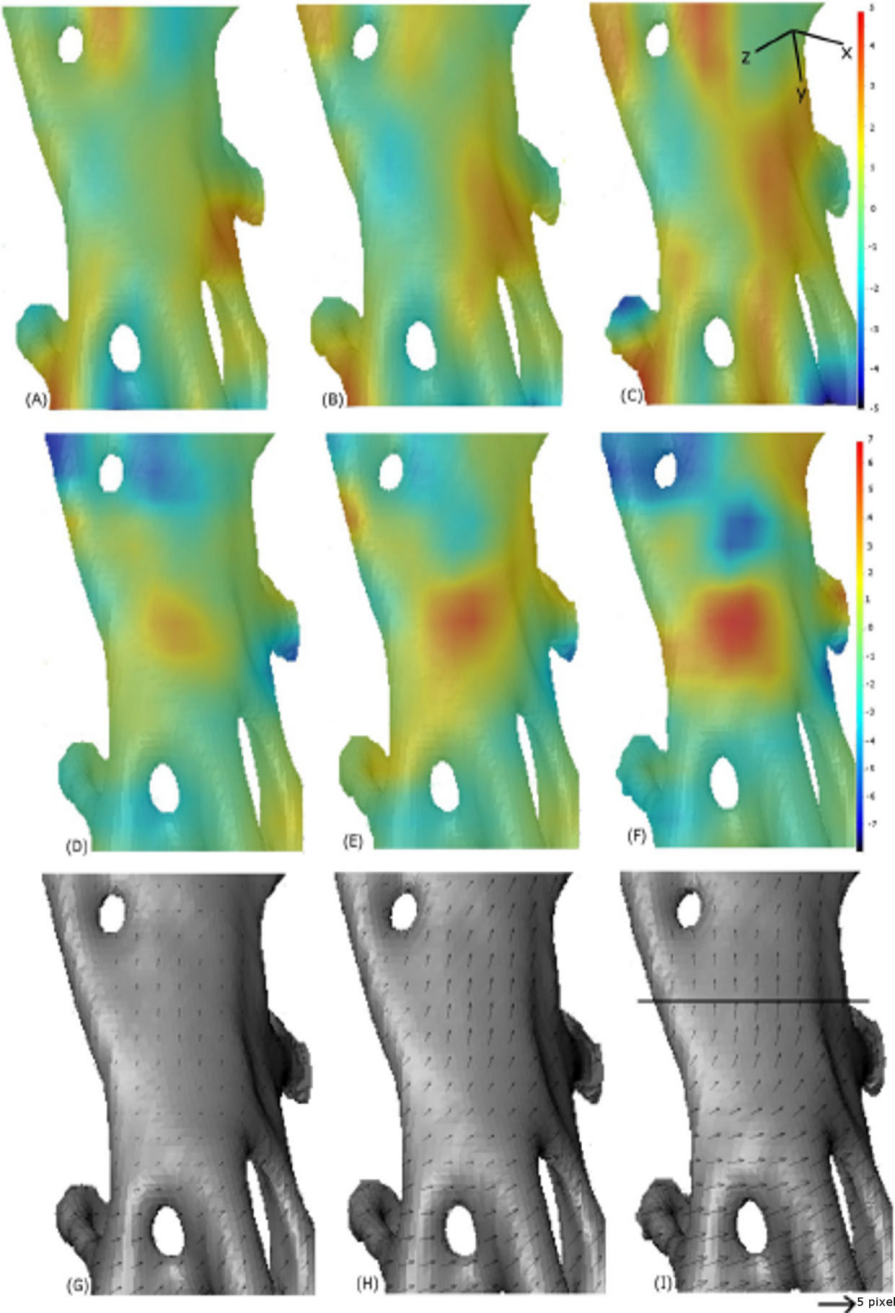
Digital image correlation. Progression of strain in *x*-direction (A–C), progression of strain in *y*-direction (D–F) and displacement maps (G–I) for scans 2, 4 and 6. The quantities are measured relative to the reference image scan 0. The convention with positive strain (red) corresponds with extension and negative (blue) corresponds with compression. The line on (I) indicates where the crack appeared, between scan 6 and scan 7. The small arrows in sub-plots G–I are displacement vectors. The scale bar is ∼0.15 mm in length.

It is not apparent from the first three scans of Fig. 12 that a crack is about to form. However, DIC enabled a more quantitative measure of the deformation process. A crack feature as shown in Fig. 12 was chosen for finer scale investigation using DIC (Fig. 13).

The crack feature appears in scan 7 (Fig. 12D) and DIC cannot therefore be used for scans 7–10. A black line in Fig. 13I shows where exactly the crack appeared in the successive scans.

The displacement vectors in (Figs. 13G–13I) show that the bottom half of this section of bone is being displaced to the right as the top half moves upwards. In the strain maps, the *y*-component increases up to a value of 6% strain (Fig. 13F) in the region where the crack occurs (Fig. 13D).

## Discussion

In this study, a *B. leucopsis* femur has been loaded to failure within a specially designed rig mounted in an X-ray computed tomography system. The scans highlighted six distinct types of deformation mechanism in the cancellous part of the bone. These were (i) cracking, (ii) thinning (iii) tearing of cell walls and struts, (iv) notch formation, (v) necking and (vi) buckling. The results highlight that bone experiences three modes of deformation including brittle (notch formation and cracking), ductile (thinning, tearing and necking) and elastic (buckling) behaviour.

The DIC study shows that the deformation is quite heterogeneous and characterised by local strain maxima. These fields may link to mechanisms that develop at a lower length scale than the X-ray tomography scan.

The relative frequency of the features, as indicated in the histogram of Fig. 11 shows that, for this particular bone, the ductile modes are more dominant than the brittle modes. By its nature bone (usually) exhibits both ductile and brittle deformation, but some bones are more brittle than others due to variety of factors including age, composition or genetics (Currey, 2003). Care must be taken in interpreting the data as there were large intervals of time (30–45 min) between each individual load increment, so that a scan could be carried out. Ductile mechanisms like creep may increase the reported proportion of ductile mechanisms over brittle ones because of the long hold times for the tomography scans. In future tomography experiments, it will be possible to carry out this type of 4D scan in real time. This will help determine whether any of the ductile deformation arose due to relaxation creep in between successive scans.

In this paper, a bird (*B. leucopsis*) femora was selected. Typically this type of bone is subject to only moderate axial loading and is ‘designed’ to withstand significant bending stress. In other vertebrates, for example mammals, the form and function of bones will be different. Although the response to loading and the relative proportion of mechanisms that occur will most likely be species specific, we expect that the type of mechanisms that occur in vertebrate bones to be similar across species.

## Conclusion

The authors have successfully designed and carried out an experiment that permits a time-lapse study of a vertebrate long bone subjected to incremental loading until failure. We have characterised the processes and mechanisms that lead to failure qualitatively using 3D X-ray computed tomography and quantitatively using DIC.

The results of this study will be of interest to a broad community of researchers who are using computer modelling to predict the load bearing capabilities of bone in various scenarios. Bone’s response to loading is complex and occurs due to mechanisms that take place at a lower length scale than the resolution of the X-ray computed tomography data. The study provides an overall insight into deformation mechanisms inside vertebrate long bones, which is not possible otherwise with the naked eye.

## Supplemental Information

10.7717/peerj.3416/supp-1 Supplemental Information 1 Specification file for CT Scanning.

## Additional Information and Declarations

### Data Availability

The following information was supplied regarding data availability: The raw data used in this study (CT scans of *Branta leucopsis* femur) are included as figures in the manuscript.
